# An assessment on the role of endophytic microbes in the therapeutic potential of *Fagonia indica*

**DOI:** 10.1186/s12941-017-0228-7

**Published:** 2017-08-01

**Authors:** Lubna Rahman, Zabta K. Shinwari, Irum Iqrar, Lutfur Rahman, Faouzia Tanveer

**Affiliations:** 1 Molecular Systematics and Applied Ethno Botany Lab (MoSEL), Department of Biotechnology, Quaid I Azam University, Islamabad, Pakistan; 2 Pakistan Academy of Sciences, 3-Constitution Avenue Sector G-5/2, Islamabad, Pakistan

**Keywords:** Natural products, Therapeutic compounds, *Fagonia indica*, Endophytic bacteria, Antioxidant, Antimicrobial, Leishmanicidal, Protein kinase

## Abstract

**Background:**

Natural products of animals, plants and microbes are potential source of important chemical compounds, with diverse applications including therapeutics. Endophytic bacteria that are especially associated with medicinal plants presents a reservoir of therapeutic compounds. *Fagonia indica* has been recently investigated by numerous researchers because of its striking therapeutic potential especially in cancer. It is also reported that endophytes play a vital role in the biosynthesis of various metabolites; therefore we believe that endophytes associated with *F. indica* are of crucial importance in this regard. The present study aims successful isolation, molecular identification of endophytic bacteria and their screening for bioactive metabolites quantification and in vitro pharmacological activities.

**Methods:**

16S rRNA gene sequencing was used for the identification of isolated endophytic bacteria. Methanolic extracts were evaluated for total phenolic contents (TPC), total flavonoids contents (TFC), DPPH free radical scavenging activity, reducing power and total anti-oxidant assays were performed. And also screened for antibacterial and antifungal activities by disc diffusion method and their MIC were calculated by broth dilution method using microplate reader. Further, standard protocols were followed for antileishmanial activity and protein kinase inhibition. Analysis and statistics were performed using SPSS, Table curve and Origin 8.5 for graphs.

**Results:**

Bacterial strains belonging to various genera (*Bacillus*, *Enterobacter*, *Pantoea*, *Erwinia* and *Stenotrophomonas)* were isolated and identified. Total phenolic contents and total flavonoids contents varies among all the bacterial extracts respectively in which *Bacillus subtilis* showed high phenolic contents 243 µg/mg of gallic acid equivalents (GAE) and *Stenotrophomonas maltophilia* showed high flavonoids contents 15.9 µg/mg quercitin equivalents (QA), total antioxidant capacity (TAC) 37.6 µg/mg of extract, reducing power (RP) 206 µg/mg of extract and 2, 2-diphenyl-1-picrylhydrazyl (DPPH) free radical scavenging activity with 98.7 μg/mL IC_50_ value. Although all the extracts tested were active to inhibit growth of selected pathogenic microbes (bacteria and fungi), but significant antibacterial activity was observed against *Klebsiella pneumonia* and *B. subtilis.* An *Enterobacter cloaca was* active against *Leishmania tropica* with IC_50_ value of 1.4 µg/mg extracts. *B. subtilis* and *Bacillus tequilensis* correspondingly exhibit significant protein kinase inhibition of 47 ± 0.72 and 42 ± 1.21 mm bald zones, indicating anti-infective and antitumor potential.

**Conclusions:**

Our findings revealed that crude extracts of selected endophytic bacteria from *F. indica* possess excellent biological activities indicating their potential as an important source of antibiotics (antifungal, antibacterial) compounds.

## Background

Drug resistance has created new health challenges for humans. Over the years, emergence of new infectious diseases such as Ebola, Swine flu (H1N1), Dengue fever and severe acute respiratory syndrome (SARS), have been added to this challenges [1]. There is a general need for chemotherapeutic agents, antibiotics and pharmaceutical drugs with high effectiveness and low cytotoxicity. New medicinal agents are also needed for nematode infections such as malaria and for the treatment of parasitic protozoan diseases such as leishmaniasis and trypanosomiasis [2].

Natural resources are the potential source of novel bioactive molecules and can be used in the treatment of various infections. Such sources may include various forms of life like plants, marine macro-organisms (sponge, corals and algae) and endophytes (fungi, bacteria and actinomycetes) [3]. Plants which are used in traditional medicines are of significant importance and therefore considerable research has been carried out on medicinal plants for bioactive compounds however limited research has been performed on the associated microorganisms. Endophytes are chemical synthesizer inside plants. They play a role as a selection system for microbes to produce pharmacologically active substances with low toxicity toward mammalians [4]. Endophytes are sometimes responsible for the medicinal properties of their hosts. Many endophytes synthesize bioactive compounds that can be used by plants for defense against pathogens and some of these compounds may be valuable as pharmaceutical drugs [5].

*Fagonia indica* is a small spiny shrub that belongs to the family Zygophyllaceae present in warm and arid regions of the world, especially Asia and Africa [6]. *Fagonia indica* is of great ethno pharmacological importance with multiple therapeutic activities such as antimicrobial, antioxidant, antiseptic, anticancer, antidiabetic and anti-inflammatory [7]. The plant is also used for the treatment of fever, thirst, vomiting, asthma, urinary discharge, dysentery, liver and stomach trouble, toothache, typhoid and skin diseases [8].

This study was designed to investigate the role of endophytic bacteria in the medicinal properties of *F. indica.* Bacteria were isolated, identified and screened for the production of bioactive secondary metabolites. All the experiments were carried out in the Molecular Systematics and Applied Ethnobotany Lab, Department of Biotechnology, Quaid-i-Azam University, Islamabad.

## Methods

### Plants collection and identification

The plant samples were collected in sample bags from village Mullazai, Peshawar, Khyber PakhtunKhwa, Pakistan and brought to the laboratory at the same day for the isolation of endophytic bacteria. The plant material was taxonomically identified as *F. indica* at the Department of plant sciences, Quaid-i-Azam University Islamabad. To further confirm the taxonomic validity of the plant species, DNA barcoding was executed using cDNA markes such as *matK*, *rbcL* and *trnH*-*psbA*. Herbarium specimen (Voucher No. MOSEL-320) was deposited in the herbarium of Molecular Systematics and Applied Ethnobotany Lab at Department of Biotechnology; Quaid-i-Azam University, Islamabad.

### Isolation of endophytic bacteria from stem of *Fagonia indica*

The stem of the plant was cut into pieces of about 0.5–1 cm in length and surface sterilized with 70% ethanol for 2 min followed by washing with Clorox (commercial bleach) for 5 min and finally rinsing at least three to five times with sterile distilled water. Stem pieces (5–6) were blotted on the blotting paper [9] and placed on selected Tryptic Soy Agar (TSA) media for the isolation of endophytic bacteria. The plates were incubated at 30 °C for 24 h to obtain colonies of bacteria.

### Molecular identification of isolated endophytic bacteria

Bacterial genomic DNA was isolated using alkaline lysis method and 16S rRNA gene was amplified by performing colony PCR using universal bacterial primers 27F and 1492R, which produced a product of size 1465 base pairs [10]. Purification of PCR product was done by using pure LinkTM Quick PCR purification Kit (Invitrogen). Sanger sequencing method was used to commercially sequence the purified PCR samples with 27F primer from Macrogen (South Korea). Sequence results obtained were compared with nucleotide sequences available on NCBI database (www.ncbi.nlm.nih.gov/BLAST) and also confirmed from EZ-taxon (http://www.ezbiocloud.net/eztaxon). Sequences of endophytic bacteria were submitted to the GeneBank (NCBI) and Accession numbers were obtained.

### Extraction of secondary metabolites

Selected bacterial strains were inoculated in Tryptic Soye Broth and incubated for 48 h in shaking incubator at 30 °C at 120 rpm. Bacteria were then transferred into 50 mL falcon tube and centrifuged at 10,000 rpm for 10 min. The supernatant was separated in the tube and the pellet was processed further. Pellet was dissolved in organic solvent (methanol) and incubated for 1 day. Next day sonication was performed to rupture the cells for 30 min after every five min. After sonication the tubes were centrifuged for 10 min at 10,000 rpm. The organic supernatant was separated in the falcon tube (A) and in the remaining pellet, again organic solvent methanol was added and centrifuged for 10 min at 10,000 rpm. Supernatant was separated in the falcon tube (B). The pellet was discarded and both the solvents (A) and (B) were mixed. The solvent was evaporated at room temperature to get crude extract of bioactive metabolites. The extract was dissolved in (dimethyl sulfoxide) DMSO [11].

### Biological evaluation

#### Total flavonoid (TF) and total phenolic contents (TPC) determination

##### Total flavonoid contents (TFC)

Aluminum trichloride (AlCl_3_) colorimetric method was used for total flavonoids content determination as described by Quettire-Dele et al. [12] with slight modifications as per system suitability. An aliquote of 20 μl of the test sample (4 mg/mL) along with negative and positive controls i.e. DMSO and quercetin (1 mg/mL) respectively were taken in 96-well plate and incubated for 30 min at 37 °C. It was followed by the addition of 10 µl of aluminum chloride (10%), 10 µl potassium acetate (98.15 g/l) and final volume was raised up to 200 µl with distilled water. Absorbance was measured at 405 nm through micro plate reader (ELx800BioTek) and triplicate results were analyzed as µg QE/mg extracts.

##### Total phenolic contents (TPC)

Folin-Ciocalteu method as described by Jagadish et al. [13] with slight modifications was followed for TPC determination. Calculated volume of test sample 20 µl was taken form 4 mg/mL stock solutions and added to 96 well plates followed by addition of 90 µl of 10 times diluted Folin-Ciocalteu reagent incubated for 5 min. After incubation, 6% sodium chloride solution was added to each mixture and incubated for 90 min at room temperature. DMSO and Gallic acid (1 mg/mL) were taken as negative and positive controls, respectively. Absorbance was measured at 630 nm wave length of triplicate analysis and results were expressed in term of mean ± SD.

### Determination of antioxidant potential of crude extracts

#### Radical scavenging activity-DPPH assay

The method described by Tai et al. [14] was used for the determination of free radicals scavenging activity with minor modification. The antioxidant potential of the crude extracts was gauged by monitoring its capacity to quench the stable 2, 2-diphenyl 1-picrylhydrazyl (DPPH) free radical. Activated crude extract samples of bacterial endophytes i.e. 100, 50, 25 and 12.5 µg/mL were taken in 96 well plates. DPPH was added to the entire row of well containing samples to obtain the final concentration of 200 µl. DMSO and ascorbic acid were taken as negative and positive controls, respectively. The absorbance was measured at 630 nm using microplate reader (ELx800 BioTek) after 1 h of incubation at room temperature. IC_50_ values expressed as µg AAE/mg of extracts. Percent radical scavenging was calculated by using formula:

$$\% {\text{RSA}} = \left[ { 1 - \left( {\text{OD of Extract}} \right)/\left( {\text{OD of Control}} \right)} \right] \times 100$$

### Total antioxidant capacity estimation

Total antioxidant assay was performed to evaluate the total antioxidant capacity of extracts by phosphomolybdenum method [15]. The method depends on the reduction of Mo (VI) to Mo (V) by the consequent formation of a green colored phosphate/Mo (V) complex with a maximal absorption at 630 nm and through antioxidant mediators [16]. Reaction mixture contains 180 µl of phosphomolybdenum reagent (0.6 M H2 SO4, 28 mM NaH2PO4, 4 mM ammonium molydate) and 20 µl of test sample taken from stock solution (4 mg/mL) followed by incubated for 90 min at 95 °C in water bath. After incubation, samples had been cooled at room temperature and transferred to 96 well plates. DMSO and ascorbic acid were taken as negative and positive controls, respectively and absorbance was measure at 630 using microplate reader (ELx800 BioTek). Results were calculated as the number of µg equivalents of ascorbic acid per mg of extract (µg/mg). The experiment was performed in triplicate.

### Total reducing power estimation

The method described by Oyaizu et al. [17] was followed for the estimation of total reducing power with minor modifications. A proper volume of test samples 40 µl from the stock solution (4 mg/mL) was taken in eppendrof tubes and incubated in water bath for 20 min at 50 °C, after the addition of reagents 0.2 M Phosphate buffer (6.6 pH) and 1% Potassium ferri cyanide [K3Fe (CN)6] (1 g 100 mL^−1^). After incubation 10% trichloroacetic acid was added to all tubes and centrifuged at 2500 rpm for 10 min. 166.66 µl supernatant from each centrifuge mixture was taken and transferred into 96 well microplate followed by the addition of 33.3 µl ferric chloride (0.1%) solution to each well and mixed properly. Absorbance was measured at 630 nm through micro plate reader (ELx800 BioTek). Results were calculated as µg AAE/mg extracts of triplicate analysis. DMSO and ascorbic acid were used as negative and positive controls, respectively.

### Antimicrobial activity

#### Antifungal assay

Triplicate analysis by disc diffusion method was used to evaluate the antifungal potential of test extracts described by Lai et al. [18] with some modification. Crude extracts were screened against four fungal pathogenic strains: *Mucor mycosis* (FCBP (Fungal culture bank of Pakistan) -0041), *Aspergillus flavus* (FCBP-0064), *Aspergillus fumigates* (FCBP-1264) and *Aspergillus niger* (FCBP-0198). Dimethyl sulfoxide (DMSO) disc was used as negative control whereas amphotericin B was used as positive control. Plates were incubated at 37 °C for 24–48 h with periodic observations of inhibition zones. Extracts were screened to determine minimum inhibitory concentration (MIC) producing an inhibition zone ≥10 mm in diameter at lower concentrations ranging from 100 to 12.5 μg/disc by standard disc diffusion method.

#### Antibacterial assay

In vitro antibacterial potential of the test extracts was evaluated using 96 well microplates method as described previously by Gao et al. [19] with slight modifications. The extracts were tested against gram positive bacteria: *Bacillus subtilis* (ATCC-6633), *Staphylococcus aureus* (ATCC-6538), *Micrococcus luteus* (ATTC-10240) and gram negative bacteria: *Escherichia coli* (ATCC-15224), *Salmonella typhi* (ATCC-14028) *Pseudomonas aeruginosa* (ATCC-9721) and *Klebsiella pneumonia* (ATCC-4619). Bacterial strains were inoculated in 10 mL TSB and incubated for 24 h at 37 °C at 120 rpm in shaking incubator. For each bacterial strain, a standardized inoculum (1 × 10^8^ CFU/mL) was prepared. The test samples were used in three fold dilutions i.e. 100, 33.3, 11.1 and 3.7 µg/mL. DMSO was used as negative control and cefixime monohydrate (antibiotic) as positive control, respectively. Reading was initially taken at zero hours and then again after 24 h incubation through microplate reader (ELx800BioTek) at 630 nm wave length [20]. The corresponding 50% inhibitory concentration (IC_50_) of each sample was calculated.

### Antiprotozoal activity

#### Antileishmanial assay

The process described by Ma et al. [21] was used for in vitro antileishmanial activity with slight modifications against *Leishmania tropica* in their promastigote stage. The parasite was sub cultured in the RPMI 1640 medium supplemented, 292 µg/mL l-glutamine, 4.5 mg/mL glucose and 10% fetal bovine serum (FBS). Within these culture conditions, the stationary phase of parasite growth was obtained in 6 days. The culture was incubated at 25 °C and used within 2 weeks of cultivation. Antileishmanial activity of extracts in comparison to amphotericin B drug was evaluated against parasite using MTT 3-(4, 5-dimethylthiazol-2-yl-2, 5-diphenyltetrazolium bromide) based micro assay as a marker of cell viability. The bacterial extract samples were tested at lower concentration with three-fold serial dilutions and relative optical density (OD) was taken at 540 nm through micro plate reader (ELx800BioTek). IC_50_ values were obtained from the dose–response curves generated by plotting percent growth versus drug concentration. Percent viable cells were calculated by using the formula.

$${\text{\% viable cells}} = \left( {\frac{{({\text{Absorbance of sample}} - {\text{Absorbance of empty well}})}}{{\left( {{\text{Absorbance of costant}} - {\text{Absorbance of empty well}}} \right)}}} \right) \times 100$$

#### Protein kinase inhibition assay

The protein kinase inhibition assay was performed thrice with purified isolates of *Streptomyces* 85E strain by observing hyphae formation using ISP4 selective media [22]. The prepared media was subjected to autoclaving for 20 min at 121 °C and poured in the petri plates (autoclaved) under Laminar flow cabinet to avoid contamination. After solidification of the media, Streptomyces culture broth was spread on the surface of media with sterilized cotton swab. The test samples 20 mg/mL were tested on this media using the disc diffusion method. Plates were incubated at 37 °C for 24–48 h. The zone formation was measured through Vernier caliper. Surfactin (antibiotic) was used as positive control while DMSO as negative control, respectively. Two types of zones were observed clear and bald in which the bald zones showed protein kinase inhibition activity [23].

### Statistical analysis

All experiments were conducted in a completely randomized design at least three times. Statistical analysis was carried out using SPSS 22.0 and Statistic 8.1. The relationship between different parameters was assessed using Pearson’s correlation coefficient (r). One-way ANOVA was used to check the significant mean difference with Tukey’s HSD for post hoc analysis. A P < 0.05 was used to define significant results. All the graphs were made using Origin 8.1 and Duncan’s multiple-range test was used to find differences among treatments (P < 0.05).

## Results

### Isolation and identification of endophytic bacteria

The isolated bacteria form *F. indica* [MOSEL (Molecular Systematic and Applied Ethno-botany Lab)-FLS1, MOSEL-FLS2, MOSEL-FLS3, MOSEL-FLS4, MOSEL-FLS5, MOSEL-FLS6, MOSEL-FLS7, MOSEL-FLS8] (5.78 × 101 CFU/mL) were identified based on 16S rRNA gene sequence analysis. The results revealed that all bacterial isolates from the stem of *F. indica* belongs to 5 different genera namely: *Bacillus, Erwinia, Pantoea, Enterobacter* and *Stenotrophomonas.* The nucleotide sequences obtained in this work were deposited in GenBank under accession numbers KT367786–KT367793 (Table 1).

**Table 1:** Identification of endophytic bacterial isolates from stem of *Fagonia indica* based on 16S rRNA partial sequences and their accession numbers in Genbank

Strain name	Closest match	Similarity % (NCBI)	Accession no
MOSEL-FLS1	*Enterobacter hormaechei*	100	KT367786
MOSEL-FLS2	*Stenotrophomonas maltophilia*	100	KT367787
MOSEL-FLS3	*Bacillus tequilensis*	100	KT367788
MOSEL-FLS4	*Erwinia* sp.	99	KT367789
MOSEL-FLS5	*Pantoea dispersa*	98	KT367790
MOSEL-FLS6	*Pantoea cypripedii*	98	KT367791
MOSEL-FLS7	*Enterobacter cloacae*	99	KT367792
MOSEL-FLS8	*Bacillus subtilis*	100	KT367793

### Biological evaluation

#### Total flavonoid (TFC) and total phenolic contents (TPC) determination

##### Total flavonoid content (TFC)

**Fig. 1:**
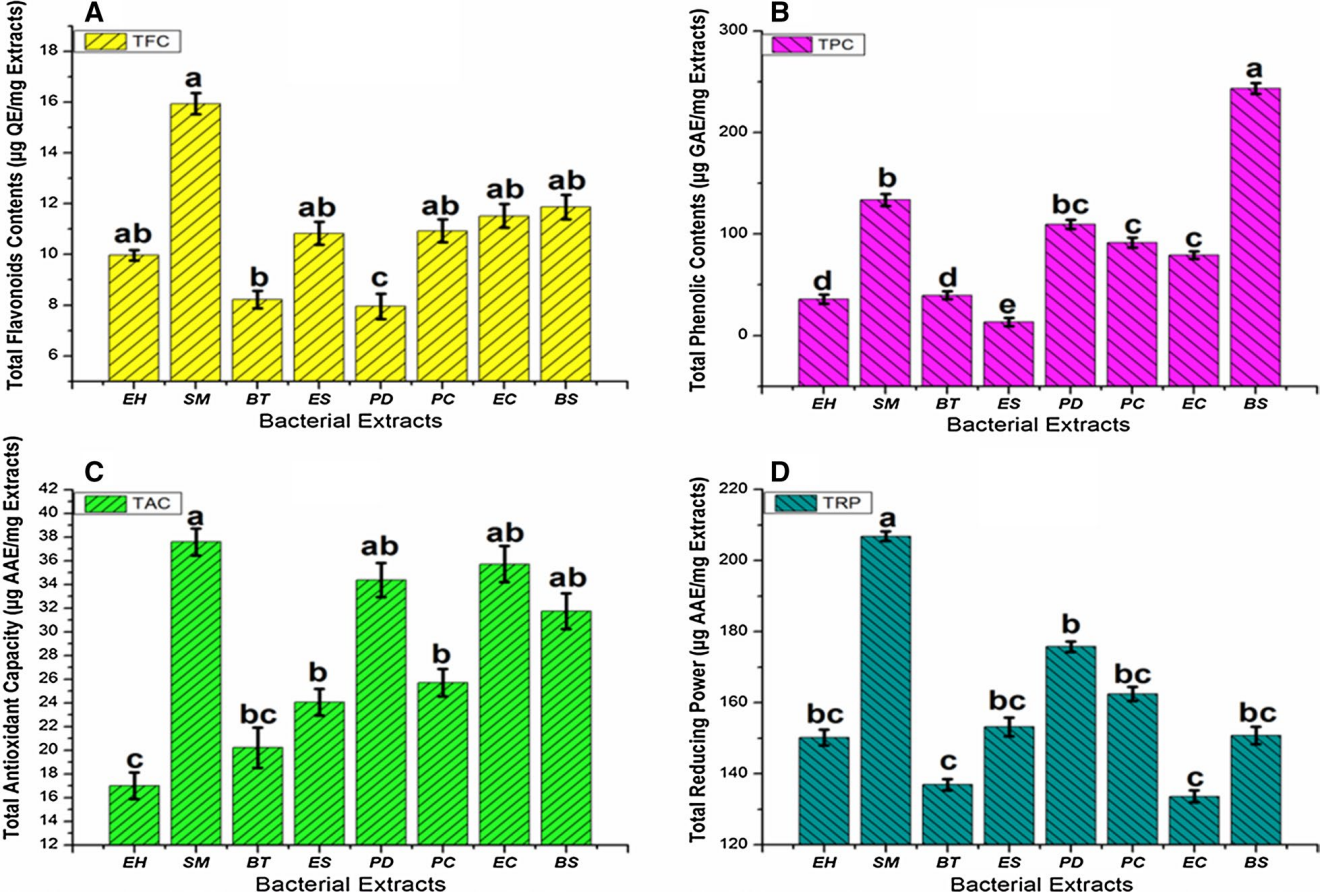
**A** TFC (total flavonoid content µg QE/mg), **B** TPC (total phenolic content µg GAE/mg), **C** TAC (total antioxidant capacity µg AAE/mg), **D** TRP (total reducing power µg AAE/mg) of bacterial crude extracts EH (*Enterobacter hormaechei*), SM (*Stenotrophomonas maltophilia*), BT (*Bacillus tequilensis*), ES (*Erwinia* sp.), PD (*Pantoea dispersa*), PC (*Pantoea cypripedii*), EC (*Enterobacter cloacae*) and BS (*Bacillus subtilis*). *Lowercase letters* compare the bacterial crude extract and control in the same concentration using one-way analysis of variance (ANOVA), followed by Tukey’s HSD (honestly significant difference). Same *lowercase letters*—no statistically significant difference (P > 0.05). *Different lowercase letters*—statistically significant difference (P < 0.05)

**Fig. 2:**
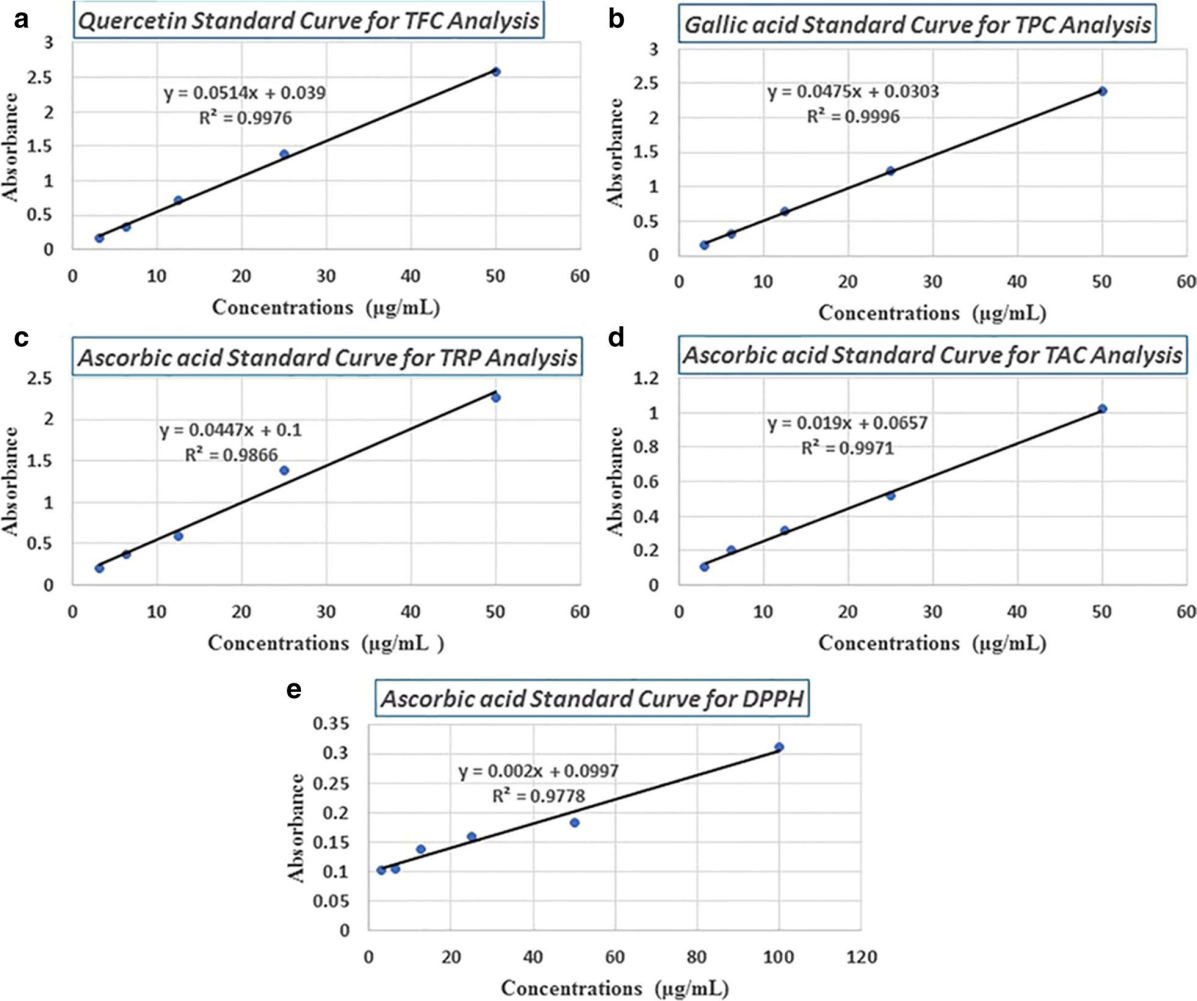
Standard curves labeled with letters from **a** to **e**. **a** Quercetin calibration curve (y = 0.0514x + 0.039, R^2^ = 0.9976) for total flavonoids contents (TFC) estimation. **b** Gallic acid calibration curve (y = 0.0475x + 0.0303, R^2^ = 0.9996) for total phenolic contents (TPC) determination. **c** Ascorbic acid calibration curve (y = 0.0447x + 0.1, R^2^ = 0.9866) for total reducing power (TRP) analysis. **d** Ascorbic acid calibration curve (y = 0.019x + 0.0657, R^2^ = 0.9971) for total antioxidants capacity determination. **e** Ascorbic acid calibration curve (y = 0.002x + 0.0997, R^2^ = 0.9778) for DPPH free radical scavenging activity analysis

The methanolic bacterial crude extracts show the presence of wide range of flavonoids contents [Figs. 1A, 2a (Standard curve)]. Among bacterial extracts *S. maltophilia* shows highest flavonoids contents of 15 µg/mg of extract followed by *Enterobacter cloacae* and *B. subtilis* with 11 µg/mg of extracts, *Erwinia* sp. and *P. cypripedii* shows 10 µg QE/mg of extract.

##### Total phenolic contents (TPC)

Bacterial crude extract shows high range of total phenolic contents. The values varied from 13 to 243 µg gallic acid equivalents per mg of extract. In our study the highest content of Gallic acid equivalent phenols was observed in extract of *B. subtilis* with 243 µg GAE/mg of extract followed by *S. maltophilia*. Whereas, *Erwinia* sp., *E. hormaechei* and *B. tequilensis* extracts contained considerably least concentration of phenols [Figs. 1B, 2b (Standard curve)].

### Determination of antioxidant potential of crude extracts

#### DPPH radical scavenging activity

The percent free radical scavenging activity (%RSA) of the bacterial crude extracts was evaluated by the discoloration of DPPH reagent. The reaction was visible as a color change from reduction of the purple colored DPPH to stable yellow-colored 2, 2-diphenyl-1-picrylhydrazyl molecule by hydrogen radical or accepting electron from donor antioxidant. Results were evaluated by calculating the half maximal (50%) inhibitory concentration. It was found that *S. maltophilia* possess highest free radical scavenging activity with 98.7 µg/mL inhibitory concentration (IC_50_ value), followed by *P. dispersa* and *E. cloacae* having 157.6 and 228.3 µg/mL IC_50_ values. Ascorbic acid was taken as standard and percent DPPH free radical scavenging activity of each endophytic bacterial extracts are shown in Table 2. [Fig. 2e (standard curve)].

**Table 2:** DPPH free radical scavenging activity of different endophytic bacteria extracts isolated from *Fagonia indica* with IC_50_ values

Bacterial methanolic extracts	400 μg/mL	200 μg/mL	100 μg/mL	50 μg/mL	IC_50_ value
*Enterobacter hormaechei*	48.48	44.34	46.07	43.40	>500
*Stenotrophomonas Maltophilia*	64.97	53.03	46.02	43.76	98.7
*Bacillus tequilensis*	52.56	45.65	50.58	44.61	334.9
*Erwinia* sp.	55.13	46.02	41.04	40.58	239.8
*Pantoea dispersa*	55.34	51.20	44.50	41.05	157.6
*Pantoea cypripedii*	50.37	45.91	47.95	42.98	467.4
*Enterobacter cloacae*	54.24	46.43	42.51	42.40	228.3
*Bacillus subtilis*	51.36	46.23	47.17	43.19	427.1

### Total antioxidant capacity

Estimating total antioxidant capacity. It was found that *S. maltophilia* show maximum antioxidant activity among all the bacterial extracts with 37 µg/mg value followed by *E. cloacae*, *P. cypripedi* and *B. subtilis* with 35, 34 and 31 µg/mg total antioxidant capacities [Figs. 1C, 2d (standard curve)].

### Reducing power

Reducing ability was measured by change of Fe^3+^ to Fe^2+^ in reducing power assay. Extracts were checked for their antioxidant reducing power. *S. maltophilia* and *P. dispersa* results indicated their electron donor ability for stabilizing free radicals and greater reductive potential with highest reducing power i.e. 206 and 175 µg ascorbic acid equivalents per mg of extract. Activity of all bacterial extracts equivalent to ascorbic acid with respect to their absorbance values were shown in Figs. 1D and 2c (standard curve).

### Antimicrobial assays

#### Antibacterial assay

In antibacterial assay, all bacterial extracts show inhibition against two strains of pathogenic bacteria *B. subtilis* and *K. pneumonia*. Against *K. pneumonia*, bacterial extracts showed IC_50_ values ranging from 1 to 20 μg/mL with variable inhibition from 55 to 82 at a concentration of (100 µg/mL). While in case of *B. subtilis* the extracts show variable IC_50_ ranging from 27 to 365 μg/mL with percent inhibition from 0 to 76. The inhibition value and effective IC_50_ value of different endophytic bacterial extracts against *K. pneumonia* and *B. subtilis* were shown in (Table 3).

**Table 3:** Percent (%) inhibition of methanolic extracts of endophytic bacteria against *K. pneumonia* and *B. subtilis* at 100 µg/mL concentrations and their IC_50_ values

Bacterial extracts	*Klebsiella pneumonia*		*Bacillus subtilis*	
	% inhibition value	IC_50_ value	% inhibition value	IC_50_ value
*E. hormaechei*	75.23	1.19	34.01	365
*S. maltophilia*	71.21	4.5	76.41	27
*B. tequilensis*	82.0	1.8	53.42	83
*Erwinia* sp.	74.23	1.21	53.31	73
*P. dispersa*	55.09	3.8	50.30	72
*P. cypripedii*	66.32	1	39.32	>100
*E. cloacae*	70.31	2.4	14.29	>100
*B. subtilis*	65.30	20	0	>100

#### Antifungal assay

**Fig. 3:**
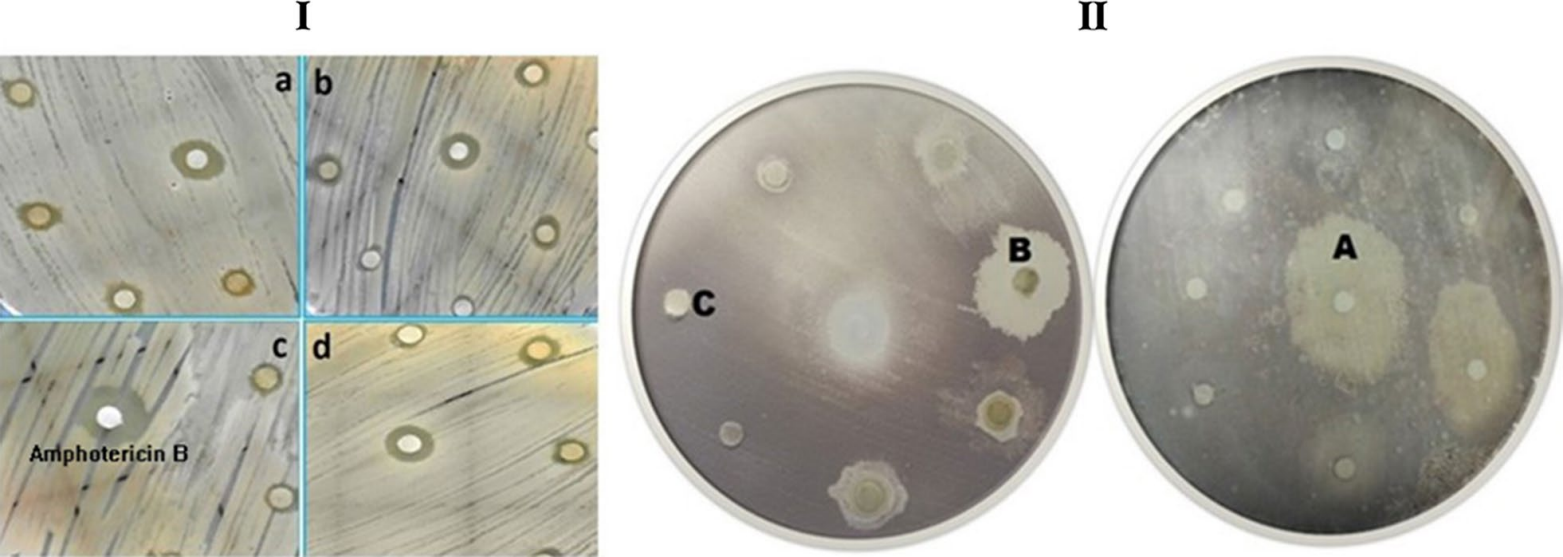
Section labeled as “**I**” shows antifungal activity of bacterial crude extracts against pathogenic strains (*a*) *Aspergillus niger*, (*b*) *Mucor mycosis*, (*c*) *Aspergillus fumigatus*, (*d*) *Aspergillus flavus*. Section “**II**” indicates protein kinase inhibition activity of bacterial extracts using streptomyces strain as model organism (*A* represent bald zone, *B* clear zone and *C* no zone)

Antifungal activity of bacterial crude extracts was determined against four strains of filamentous pathogenic fungi namely; *Mucor mycosis*, *Aspergillus flavus*, *Aspergillus fumigatus* and *Aspergillus niger* through disc diffusion method. All the bacterial genera showed inhibitory activity against all the selected pathogenic fungi. Almost all the bacterial extracts were active against *A. niger* but significant results observe are of *Stenotrophomonas maltophilia* with 16 mm inhibitory zone and 12.5 μg/mL MIC value (Table 4; Fig. 3). Although best results showed by *B. tequilensis* extracts against *M. mycosis* with maximum zone of 12 mm and 50 μg/mL MIC whereas all the extracts shows moderate antifungal activity against *A. fumigatus* with an average diameter of growth inhibition zone ranging from 7 to 10 mm. Moreover, no inhibitory zone was observed for DMSO which conform its inactivity against the selected fungal strains. Although standard drug amphotericin B Exhibited maximum, activity with 21.2 ± 0.985 mm zone.

**Table 4:** Antifungal activity of bacterial crude extracts against pathogenic fungi and their zone of inhibition including the diameter of disc (5 mm), the sample size was 25 μg/mL per disc (5 μl) in disc diffusion assay

Bacteria crude extracts	Pathogenic fungi and growth inhibition zone*							
	*M. mycosis*	MIC µg/mL	*A. flavus*	MIC µg/mL	*A. fumigatus*	MIC µg/mL	*A. niger*	MIC µg/mL
*Enterobacter hormaechei*	7.06 ± 0.846^a^	100	8.81 ± 1.24^a^	100	7.63 ± 1.17^a^	100	9.92 ± 0.93^a^	100
*Stenotrophomonas maltophilia*	9.12 ± 1.06^a^	100	10.58 ± 1.36^b^	100	10.04 ± 0.55^a^	100	16.74 ± 0.31^c^	12.5
*Bacillus tequilensis*	12.2 ± 1.48^a^	50	7.96 ± 0.86^a^	100	9.54 ± 0.70^a^	100	9.69 ± 0.84^a^	100
*Erwinia* sp.	6.37 ± 0.63^a^	100	6.25 ± 0.58^a^	100	7.86 ± 1.08^a^	100	7.27 ± 0.47^a^	100
*Pantoea dispersa*	9.05 ± 0.931^a^	100	6.46 ± 0.84^a^	100	7.8 ± 1.13^a^	100	11.08 ± 0.47^a^	50
*Pantoea cypripedii*	7.31 ± 1.21^a^	100	7.6 ± 0.59^a^	100	8.1 ± 1.39^a^	100	7.39 ± 0.55^a^	100
*Enterobacter cloacae*	10.2 ± 1.24^a^	100	11.10 ± 0.88^b^	50	10.12 ± 0.72^a^	100	6.14 ± 0.78^a^	100
*Bacillus subtilis*	10.1 ± 1.81^a^	100	7.24 ± 0.65^a^	100	9.08 ± 0.82^a^	100	8.11 ± 0.29^a^	100
Controls (amphotericin B)	21.2 ± 0.985	–	14.07 ± 1.07	–	29.77 ± 1.08	–	16.30 ± 1.75	–

Letter a–c represent; a highly significant, b slightly significant and c non-significant difference from control at P < 0.05 by one-way ANOVA in the column

* Values are mean ± SD of triplicate

### Antiprotozoal activity

#### Antileishmanial assay

Antileishmanial activity against promastigote was determined by MTT assay. Variation was observed between bacterial extracts (Table 5) activities against promastigote stage of *L. tropica* with IC_50_ values ranging from 1.4 to 6.6 μg/mL with different concentration. *E. hormaechei* has high inhibition with IC_50_ value of 1.4 followed by *P. cypripedii* and *B. tequilensis* with IC_50_ values of 1.5 and 1.6 µg respectively.

**Table 5:** Antileishmanial activity of bacterial crude extract against promastigotes of Leishmania

Bacterial methanolic extracts	521 µg/mL	160 µg/mL	130 µg/mL	65 µg/mL	IC_50_ value
*E. hormaechei*	31.61	32.46	37.20	37.83	2.0
*S. maltophilia*	33.02	34.32	35.80	42.51	6.6
*B. tequilensis*	34.20	37.23	38.21	40.51	1.6
*Erwinia* sp.	37.22	39.23	41.32	43.42	5.1
*P. dispersa*	36.83	38.23	40.21	42.34	2.7
*P. cypripedii*	35.52	38.41	40.32	40.32	1.5
*E. cloacae*	35.21	36.01	39.32	40.40	1.4
*B. subtilis*	35.02	36.51	38.23	41.36	2.3

#### Protein kinase inhibition assay

In the current study, endophytic bacterial crude extracts were screened for the inhibition of protein kinases with *Streptomyces* sp. The growth inhibition of *Streptomyces* strain was used as parameter to check the cytotoxicity of endophytic crude extracts. Clear zone indicates no bacterial growth having cytotoxicity while a bald zone with doted bacterial growth indicates protein kinase inhibition. In bald zone, only the hyphae formation capability of Streptomyces was lost. In clear zones, complete cells were destroyed. Bacterial extracts of genus *Bacillus* show high inhibition value against *Streptomyces* growth such as *B. subtilis* and *B. tequilensis* with 47 ± 0.72 and 42 ± 1.21 mm zone of inhibition, respectively with bald zones formation which show protein kinase inhibition activities DMSO show no-toxic effect with no zone formation while positive control surfactin show the bald zone formation with 21 ± 1.02 mm (Table 6; Fig. 3).

**Table 6:** Protein kinase (PK) inhiation (i.e. zone’s diameter in mm ± SD) during *Streptomyces* hyphae formation of isolated bacterial extracts (bald zone indicates PK inhibition while clear zone shows cytotoxicity)

Bacterial extracts	Protein kinase inhibition zones mm ± SD	
	Bald zone	Clear zone
*E. hormaechei*	–	17 ± 0.92^b^
*S. maltophilia*	–	12 ± 1.04^a^
*B. tequilensis*	42 ± 1.21^a^	–
*Erwinia* sp.	–	14 ± 1.10^a^
*P. dispersa*	–	18 ± 0.81^b^
*P. cypripedii*	–	12.99 ± 1.01^a^
*E. cloacae*	–	12 ± 1.40^a^
*B. subtilis*	47 ± 0.72^a^	–
Surfactin	21 ± 1.02	
Amphotericin B	–	17.84 ± 1.8

Surfactin and amphotericin B are positive controls

Values are mean ± SD of triplicate analysis

Letter a represent significant difference while b represents non-significant difference from their respective controls at P < 0.05, by one-way ANOVA in the column

## Discussion

A large number of bioactive metabolites with pharmacological properties have been isolated from medicinal plant’s endophytes and structurally illustrated by employing various conventional as well as modern techniques. These metabolites have provided a base line for the researchers to do more work on development and formulation of bioactive compounds into useful therapies with tremendous applications in health care system and also in many other fields of human life.

Bacteria are common inhabitants of both internal and external tissues of most plants [12]. Medicinal plants usually harbor endophytes related with their secondary metabolites and medicinal activities [5]. The present study was conducted on endophytic bacteria, isolated from *F. indica* with great ethno-botanical significance. It is reported that medical uses of a plant with medicinal history relates more to its endophytic population than its own biochemistry [5]. Many biological assays were conducted to investigate whether bacteria associated with *F. indica* have potential medicinal properties. The isolated endophytic bacteria belong to diverse genera such as *Bacillus*, *Enterobacter*, *Pantoea*, *Erwinia*, *Stenotrophomonas* as confirmed with 16S rRNA gene sequence analysis.

It was observed from the results that all the selected bacterial extracts exhibit various anti-bacteria, anti-fungal and protein kinase inhibition activities. Antioxidants are the first line of defense against damage that may occur due to the generation of free radicals. Antioxidants deactivate or stabilize free radicals before they attack the cells [24].

The free radical DPPH scavenging activity model is a simple, rapid and classic method of assessing antioxidant activity [25]. Methanolic extracts of endophytic isolates were able to reduce the stable radical DPPH to a yellow-coloured diphenyl picryl hydrazine. Among others, the culture extracts of *S. maltophilia* and *P. dispersa* showed the highest % scavenging activities which is considerable as compared to control (i.e. Ascorbic acid equivalent). IC_50_ value is a widely used parameter for the measurement of free radical scavenging activity. Low IC_50_ indicates significant activities as compared to high IC_50_ value [26]. The current results are in agreement with the previous study reported by [27], about endophytic bacteria associated with ethno-medicinal plants.

Oxidation in biological system is natural phenomenon resulting in the generation of highly reactive peroxyl and hydroxyl radical which may cause inadequate damage to DNA, polyunsaturated fatty acid residues of cell membrane, phospholipids and proteins. It may also lead to pathological effects such as cancer and vascular diseases [28]. Among the extracts total antioxidant activity of *S. maltophilia* was the highest followed by *E. cloacae* strain.

Reducing ability of a compound depends on the free radical scavenging capacity and electron donation [29]. Endophytic bacteria isolated from *Centella asiatica*, *Pantoea* species such as *Pantoea agglomerans* showed greater reductive potentials reported by Rafat et al. [30]. All bacterial extracts were subjected for their reducing power but *S. maltophilia* and *P. dispersa* showed the highest values indicating their electron donating ability for stabilizing free radicals and showed the similar reductive potential as reported above.

High antioxidant potential is usually related with higher proportion of the phenolic content and in the present study a significant correlation was also found in case with *S. maltophili.* The current results are in agreement with the previous study reported by Swarnalatha et al. [31].

Flavonoids have an important role in stabilizing lipid oxidation which is associated with antioxidant activity [32]. In present study highest flavonoid content was displayed by *S. maltophilia* culture extract which correlates with its highest radical scavenging and greatest reductive potential as discussed earlier. Which are in agreement with the study reported by Jalgaonwala et al. [33] about endophytic bacteria associated with host plant *Pongamia glabra*.

All isolated of endopytes extracts from *F. indica* showed varying degree to inhibited test organism growth but significant results were observe against *K. pneumonia* by each extracts. Bacterial extracts of *S. maltophilia*, *E. hormaechei*, *B. tequilensis* and *Erwinia* sp. also showed significant antibacterial activity against *B. subtilis*, with variable inhibition and IC_50_ values. The crude extract of isolated *B. subtilis* strain showed no growth inhibition against the *B. subtilis* which show their similar metabolites productions and thus have no effect on test strain.

Moreover, extracts of all the endophytic bacteria inhibited the growth of nearly all the tested fungal pathogens. In current study the extracts producing a growth inhibitory zone ≥10 mm in agar disc diffusion assay were considered significant active. Significant result was observed by *S. maltophilia* against *A. niger* with 16 mm inhibitory zone and 12.5 μg/mL MIC value and *B. tequilensis* extracts against *M. mycosis* with maximum zone of 12 mm and 50 μg/mL MIC. Which is an agreement with the previous results reported by [34] Non-toxic effect of DMSO was confirmed through absence of growth inhibition zones while standard drug amphotericin B exhibited inhibitory zone of (21.2 ± 0.985).

Leishmaniasis, is a vector- borne disease caused by obligate intra-macrophage protozoa [35]. More investigations required to find anti-leishmanial effect by using *Leishmania* amastigote through different in vitro assays and then to investigate in vivo activity in laboratorial infected animals which would help in obtaining a novel drug that could potentially be cost-effective against the *leishmania* parasite and less toxic [36]. Most of the isolated endophytic bacterial crude extracts showed antileishmanial activities against *leishmania tropica* with IC_50_ values ranging from 1.4 to 6.6 μg/mL.

Protein phosphorylation at threonine/serine and tyrosine residues by protein kinases is one of the major regulatory mechanisms in biological processes including cell proliferation, cell differentiation metabolism and apoptosis. Deregulated phosphorylation at serine/threonine and tyrosine residues by protein kinases produced as a result of genetic alterations acquired early in tumorigenesis are often the cause of cancer. In this respect, inhibition of protein kinases has emerged as a promising target for cancer treatment [37]. Using streptomyces 85E as an assay strain for kinase inhibitors appears to identify a wide range of eukaryotic kinase modulators, presumably because the Streptomycete enzymes are evolutionary forerunners of their highly specific eukaryotic counterparts [38].

The growth inhibition of *Streptomyces* strain was used as parameter to check the protein kinase inhibition of the crude extracts. Bacterial extracts of genus *Bacillus* showed high inhibition zone against Streptomyces growth. *B. subtilis* and *B. tequilensis* showed bald zone formation (with 47 ± 0.72 and 42 ± 1.21 mm zone respectively), positive control Surfactin also exhibit bald zone which indicate protein kinase inhibition activities. Extracts were used in concentration of 100 µg/mL while positive control with 25 µg/mL. *B. subtilis* is the source of surfactin antibiotic production as isolated and reported by [39]. Protein kinase activity is critical for the aerial hyphae formation of Streptomyces and this prerequisite has been exploited in the present study to bioprospect the extracts for their kinase inhibitory activity so that their anticancer potential could be assessed.

## Conclusions

From the preliminary study, it can be inferred that endophytes play a pivotal role in the medicinal activities of plants such as *F. indica.* We conclude excellent biological activities for the endophytic microorganisms associated with *F. indica* and postulate a viable role of the endophytic microbes in the medicinal potential of *F. indica*. We further recommend studies on the endophytic microbes associated with *F. indica* with state of the art spectroscopic and chromatographic techniques for the identification of targets and mechanism of their synthesis.
